# Response to Medical Assistance in Dying, Palliative Care, Safety, and Structural Vulnerability

**DOI:** 10.1089/jpm.2023.0581

**Published:** 2023-12-05

**Authors:** Romayne Gallagher, Ramona Coelho, Philippe D. Violette, K. Sonu Gaind, Harvey Max Chochinov

**Affiliations:** ^1^ Division of Palliative Care, University of British Columbia, Vancouver, British Columbia, Canada.; ^2^ Independent Practitioner, London, Ontario, Canada.; ^3^ Department of Surgery, Evidence and Impact, McMaster University, Hamilton, Ontario, Canada.; ^4^ Department of Health Research Methods, Evidence and Impact, McMaster University, Hamilton, Ontario, Canada.; ^5^ Department of Psychiatry, Sunnybrook Health Sciences Centre, Toronto, Ontario, Canada.; ^6^ Department of Psychiatry, University of Toronto, Toronto, Ontario, Canada.; ^7^ Department of Psychiatry, University of Manitoba, Winnipeg, Manitoba, Canada.; ^8^ Cancer Care Manitoba Research Institute, Winnipeg, Manitoba, Canada.

**Keywords:** active, assisted, editorial [publication type], euthanasia, palliative care, suicide, voluntary

## Abstract

This report, signed by >170 scholars, clinicians, and researchers in palliative care and related fields, refutes the claims made by the previously published *Medical Assistance in Dying, Palliative Care, Safety, and Structural Vulnerability*. That report attempted to argue that structural vulnerability was not a concern in the provision of assisted dying (AD) by a selective review of evidence in medical literature and population studies. It claimed that palliative care has its own safety concerns, and that “misuse” of palliative care led to reports of wrongful death. We and our signatories do not feel that the conclusions reached are supported by the evidence provided in the contested report. The latter concluded that the logical policy response would be to address the root causes of structural vulnerability rather than restrict access to AD. Our report, endorsed by an international community of palliative care professionals, believes that public policy should aim to reduce structural vulnerability and, at the same time, respond to evidence-based cautions about AD given the potential harm.

Accurate interpretation of research findings is crucial for translating knowledge and has implications for future studies, policies, and clinical practice. The interpretation and faithfulness to the original text and how a piece of evidence applies to an argument can generate what has increasingly been called “spin.”^1^ Spin has various definitions, but in biomedical literature it is “specific reporting that fails to faithfully reflect the nature and range of findings, and that could affect the impression that the results produce in readers.”^2^

We wish to express our deep concern about the flawed scholarship in the report on *Medical Assistance in Dying, Palliative Care, Safety, and Structural Vulnerability* by Downar et al.^3^

We recognize that Medical Assistance in Dying (MAiD) is a fiercely contentious and polarizing issue. We believe individuals should be allowed to voice their opinions, no matter what they are. However, we take exception to seeing those opinions presented as if they were evidence based when the conclusions appear to be reached by ignoring critical information within cited sources and engaging in speculation without basis. The resultant conclusions can only be described as spurious ones. Given its potential to affect future research, medical practice, policies, and public health, this special report cannot enter the public record without it being strongly contested.

## Withholding Critical Information

The authors point out that assisted death (AD) is more common in people with higher income and education, with the implication being that these people are not structurally vulnerable. Of the four articles referenced, two are Canadian studies conducted before AD was available to people who did not have a reasonably foreseeable death and who were more likely to be structurally vulnerable. The American study consists of 114 patients over the first two years of AD in a system where applicants must be within six months of death. The Swiss population-based study is from 2003 to 2008, with only 20 of the 1301 ADs being for mental illness.

Studies of patients whose death is not reasonably foreseeable reveal a different picture. A Dutch review of the publicly available information on 66 ADs for mental illness between 2011 and 2014 reported the ratio of women to men as 2.3:1.^4^ This statistic parallels the 2:1 ratio of females to males in attempting suicide during vulnerable times, most of whom do not attempt suicide again. The obvious concern is whether AD for mental illness is providing a lethal means for women to die, during times of vulnerability, who would otherwise recover and regain the desire to live.

Most of the disorders were depression (55%), but 12% had psychotic features, and 52% of the 66 cases had personality problems, some without an official diagnosis. The majority of patients (56%) reported unresolved social suffering including social isolation or loneliness. The majority of patients had long histories with mental illness and had tried multiple therapies, though 56% had refused some therapies making the judgment of treatment futility challenging. It must be noted that Canadian law does not require any treatments be tried or accessible before providing AD.

## Selectively Representing Data to Confirm a Point of View

The authors state that people with frailty and organ failure, whom they describe as more vulnerable and dependent than patients with cancer, receive less palliative care and do not make up nearly as many patients receiving AD. Hence, they conclude that AD is not targeting those with structural vulnerability. The article they reference by Teno et al.^5^ examines the association of functional decline comparing cancer deaths with those caused by heart failure, stroke, and diabetes.

Although people with noncancer illnesses started their last year of life with more disability than those with cancer, the latter experienced a more precipitous decline in activities of daily living (ADL) starting approximately five months before death, leading to functional impairment exceeding that of noncancer illnesses in the final months. This rapid decline was associated with where the person died and whether palliative care was involved in their last months of life.

Studies show that people with cancer receive more palliative care relative to frailty or organ failure in multiple countries, because of the “decline predictability”^6^ of cancer as opposed to the unpredictable course for organ failure and frailty.^7,8^ Hence, cancer is the most prevalent diagnosis for AD because its terminal course is predictable, and it is the most common cause of death in Canada.^9^

## Excess Mortality: Speculation Without Understanding

The authors note that life expectancy has grown in countries that permit AD as opposed to those that do not. Life expectancy in a country depends on public health outcomes resulting from policies in health care and beyond, which affect the social determinants of health.^10^ Early childhood deaths have had the most impact over the centuries, with the impact of AD in adults being minimal. It is not logical to make conclusions about one factor in complex systems in different countries.

The authors state that during COVID-19, which targeted structurally vulnerable people, AD increased even further in Canada, Belgium, and the Netherlands, yet these countries had substantially lower excess mortality during this time. Tracking excess mortality requires researchers to know the cause of death, and only some provinces in Canada mandate that AD be noted on the death certificate.^11^ As such, in the Netherlands, Belgium, and parts of Canada, it would be impossible to sort these deaths out from others to characterize them as excess mortality. Excess mortality is difficult to detect even with the best design, but especially during a pandemic, when there is another source of excess deaths that was much more common.

The authors state that the importance of these population studies “cannot be overstated.” They even double down by suggesting that a rise in life expectancy in countries allowing AD suggests vulnerability may offer protection. The evidence they provide does not support this.

## Suggesting Palliative Care Is Culpable for Unintended Deaths

The authors imply that, like AD, palliative care is culpable for unintended deaths.

The first claim notes that palliative care providers regularly advocate for improved access to opioids for people with advanced illness, and “Increased opioid availability is a key facilitator of good PC, but it is also a contributor to the opioid crisis in many countries.” They state, “Roughly 8000 Canadians died of an opioid overdose in 2021 alone; a substantial proportion of these overdoses involved a pharmaceutical opioid that was either prescribed or diverted.” They reference a Health Canada website publication that is no longer directly available, as it is updated yearly.

In the version accessible now,^12^ a large headline proclaims, “toxicity of supply continues to be a major driver of the crisis” and notes, “Of all accidental apparent opioid toxicity deaths in 2022 (January–December), 79% involved opioids that were only non-pharmaceutical.” More studies are reporting an increase in overdoses from illegal opioids, even as the use of prescription opioids is declining.^13^ Implying that palliative care is partly responsible for the opioid crisis is a misreading of the evidence and disregards the complex nature of the problem.

The second claim involves the Liverpool Care Pathway (LCP). Downar et al. state there were “numerous complaints of widespread abuse and hastened deaths, particularly among the structurally vulnerable.” The LCP independent review reported that “some relatives and carers have reached the conclusion that ‘the pathway’ represents a decision on the part of clinicians, in effect, to kill their dying patients, when that is clearly not the case.”^14^

## Do Not Listen to Any Media Cases: They Are All One Sided

The authors cast doubt on media stories showing that vulnerable people have been approved for AD. They review only two of the numerous media-reported cases that have surfaced in Canada. The first is a woman with multiple chemical sensitivities who requested and received AD. Downar et al. state her situation was incorrectly portrayed in the media as poverty and a lack of adequate housing rather than intolerable suffering related to her underlying condition. They reference testimony from her AD provider, reading a note from the woman thanking her for believing in her suffering and granting her AD.^15^

This same AD provider signed a letter to federal housing and disability government officials confirming that her symptoms improved in cleaner air environments and asked for help to find or build a chemical-free residence. Media^16^ quotes the letter saying, “We physicians find it UNCONSCIONABLE that no other solution is proposed to this situation other than medical assistance in dying.”

The second case is a man whose request for AD was because of impending homelessness. They quote a twitter post from the man saying the story was “hijacked by the right trying to spin it into their own agenda.” The full quote says: “It seems my story has been hijacked by the right trying to spin it into their own agenda. I hope people don't get distracted by this. The real issue isn't whether or not someone like me should be able to access MAiD. It's making life bearable enough that they won't need to.” We agree people should receive adequate support to live, and we desire adequate safeguards to prevent life termination when structurally vulnerable people are despairing from a social failure of society.

In both these cases, the authors edited the patient's stories and words to affirm their argument that there are no concerns of structural vulnerability driving AD.

There are some credible well-investigated cases in the media that should concern everyone.^17–19^

A 2021 Canadian academic study questioning whether unmet needs were driving requests for AD interviewed 20 of 120 clinicians of the national association of AD assessors and providers.^20^ They did this study the same year that Canada first approved AD for persons without a foreseeable death, so few cases would be expected. Although the article says unmet needs were rare, it reports “There were some cases in which the provider was worried that unmet needs were driving the request for MAiD. These situations included poor quality or inappropriate housing, inadequate home care in someone who refuses to go into long term care, long waitlists for publicly funded multidisciplinary chronic pain clinics and no local care available requiring unacceptable travel.”

The same study quoted an AD provider's take on the conflict of an applicant with structural vulnerability requesting AD from them: “I think we've become—I don't know if comfort is the right word, but we've become more accepting that there are patients that have unmet needs that if they were met may change their request or their timeline, but that those needs are not meetable under the current system, and therefore, we go ahead and approve the patient and sometimes provide MAiD.”

There is evidence in scholarly publications^20,21^ that structural vulnerability may be driving some cases of AD. Yet, in a report from 2022 testimony to a parliamentary committee reviewing AD,^22^ the lead author of the article we contest stated: “there is absolutely no data suggesting that the practice of MAiD at this point is driven to any degree by poor access to palliative care, socio-economic deprivation or any isolation.” Independent of the total numbers of confirmed cases of Canadians receiving AD for structural vulnerability, which may be under-reported given current data reporting requirements that are based on provider self-reported data rather than prospective oversight, we are concerned by the blanket dismissal and minimization of any deaths by AD for unmet social needs.

## Oversight of AD: Only What Looks Good

The article states: “Any concerning cases are referred to the appropriate regulatory body for further investigation. Although we do not have reports from all jurisdictions…”.^3^ It references the Quebec oversight body that publishes a yearly report. No details about cases who did not comply with legislation, nor what was done about it are provided. Dr. Michel Bureau, head of the oversight body, recently sent a memo to doctors that was published online,^23^ reminding them to stay within the limits of the law.

Two notes from the memo stand out: “Advanced age and aging-related problems are not a serious illness and incurable and do not warrant MAiD”; and “Shopping around” for a favorable second opinion is not an acceptable practice.” Dr. Bureau stated during an interview^24^ that 15 out of 3663 doctor-ADs in Quebec between spring 2021 and spring 2022 did not comply with the law. The physician's regulatory body declined to take any disciplinary action.

In Ontario, Canada's most populous province, the Ontario coroner reviews AD deaths, and the only public document that appears is their framework of notification of assessors/providers if they are not following regulations. It appears to require ongoing violations before any reporting to a regulatory body or police occurs.^25^ Thus, it is not surprising that no cases related to AD have yet to appear in the Ontario College's case outcomes. The chief coroner of Ontario recently announced a Medical Assistance in Dying Death Review Committee (MDRC)^26^ in response to increasing health, social, and intersectional complexities arising from current and pending legislative changes. The MDRC will provide an independent expert review of MAiD deaths to assist in evaluating public safety concerns.

British Columbia (BC) has a MAiD Oversight Unit that lies within the ministry of health, but it has no publications or public facing website. There is only one BC coroner's death review panel on AD and that covers only the year 2016.^27^ The report noted regional variations in the provision of AD and a lack of a framework for case review. In 2018, the coroner's office announced that the only AD cases they would be reviewing were those where the underlying condition leading to the request for MAiD relates to an accident, violence, or self-inflicted injury; or if the death was provided in a mental health or correctional facility.^28^

The memo from the Quebec oversight body and the formation of the Ontario MDRC have happened since the publication of the article we are contesting. These changes counter the article's assertions that all is well and that we should trust the current oversight.

## Conclusion: More Spin

The article closes by restating the major argument that the data show no evidence of structural vulnerability in the provision of AD and that controversies in palliative care are the mirror image of those with AD. The evidence, however, simply does not support the many claims and conclusions.

There is evidence of people receiving AD when they are structurally vulnerable, and providing death to people for those situations was never intended by the legislation and should be rigorously and transparently investigated. We believe that public policy should aim to reduce structural vulnerability in all people and, at the same time, be responsive to evidence-based cautions about AD given the potential harm. We take no pleasure in writing this rebuttal but feel compelled to expose what appears to be spin that is harmful to future scholarship, clinical practice, health policy, and public education focused on the practice of assisted dying.

## An International Community of Palliative Care Professionals

**Tristan Anderson, MD, FRCPC,** Psychiatrist, Manitoba, Canada; **Jeremy Bannon, MD, CCFP,** Hospitalist, Hotel Dieu Shaver, Associate Clinical Professor (Adjunct), Michael G. DeGroote School of Medicine, McMaster University, MA Candidate (Clinical Ethics), Pellegrino Center for Clinical Bioethics, Georgetown University, Ontario, Canada; **Samia Barakat, MD,** Professor Emeritus, Psychiatry, University of Manitoba, Manitoba, Canada; **Paul Barré, MD, FRCP(C),** Quebec, Canada; **Sasha Bernatsky, MD, FRCPC, PhD,** Quebec, Canada; **Eileen Biggs, MD,** Ontario, Canada; **Thomas Bouchard, MD, FCFP,** Alberta, Canada; **Eduardo Bruera, MD,** FT McGraw Chair in the Treatment of Cancer, Chair, Department of Palliative, Rehabilitation, & Integrative Medicine, MD Anderson Cancer Center, Texas, USA; **Althea Burrell, BASc, MD, FRCPC,** Adult Respirology, Ontario, Canada; **Ira Byock, MD, FAAHPM,** Emeritus Professor of Medicine and Community & Family Medicine Dartmouth Geisel School of Medicine, Montana, USA; **Casimiro Cabrera Abreu,** Associate Professor of Psychiatry, Department of Psychiatry, Providence Care Hospital, Queen's University, Kingston, Ontario, Canada; **Luigi A. Castagna, MD, FRCP(C),** Paediatric Neurology, Developmental Paediatrics, Ontario, Canada; **David Chan, MD, MSc,** Professor Emeritus, Family Medicine, McMaster University, Ontario, Canada; **David Chen, MD, MS-Bioethics DFAPA,** Maryland, USA; **Maria Cigolini, MBBS (USyd), FRACGP, FAChPM, Grad.DipPallMed(UMelb),** Senior Palliative Medicine Physician, Board Member, International Association of Hospice and Palliative Care, Associate Professor, New South Wales, Australia; **Stephen R Connor, PhD, LP,** Executive Director, Worldwide Hospice Palliative Care Alliance, London, United Kingdom; **Margaret M Cottle, MD, CCFP (PC), LM,** Clinical Assistant Professor, Division of Palliative Care, Faculty of Medicine, University of British Columbia, British Columbia, Canada; **Paul Dagg, MD, FRCPC,** Clinical Professor UBC, British Columbia, Canada; **Douglass Dalton, FCFP,** Quebec, Canada; **Jane Dobson, MD, FCFP,** Ontario, Canada; **Sinead Donnelly, MD, FRCPI, FRACP,** Associate Professor, Aotearoa New Zealand and Ireland; **P. Drijber, MD, PhD, CCFP (F), CBOM (A),** Ontario, Canada; **David D'Souza, MD, CCFP, DTM&H, DCAPM,** Pain, Addictions, Care of the Elderly, Assistant Professor, Queen's University, Ontario, Canada; **David Paul D'Souza, MD, FRCPC,** Ontario, Canada; **Mark D'Souza, MD, CCFP(EM), FCFP,** Assistant Professor, Queen's University, Ontario, Canada; **Roy Eappen, MDCM, FACE, FRCP(C), FACP, CD, FACE,** Quebec, Canada; **Timothy Ehmann, MD, FRCPC,** Child & Adolescent Psychiatrist, Alberta, Canada; **Joel N. Eisen, MD, FRCP,** Psychiatrist, Ontario, Canada; **James M. Ellison, MD, MPH,** Pennsylvania, USA; **E. Wesley Ely, MD, MPH,** Grant W. Liddle Professor of Medicine and Critical Care, CIBS Center, Vanderbilt University Medical Center, TN Valley VA GRECC, Tennessee, USA; **Sherif Emil, MDCM, FRCSC, FACS, FAAP,** Mirella & Lino Saputo Foundation Chair in Pediatric Surgical Education & Patient and Family-Centred Care Professor of Pediatric Surgery, Surgery, and Pediatrics, Associate Chair for Education & Departmental Citizenship, Department of Pediatric Surgery, McGill University Faculty of Medicine, Director; **Harvey E. Beardmore,** Division of Pediatric Surgery, Senior Investigator, The Research Institute of the McGill University Health Centre, Quebec, Canada; **Karen Ethans, MD, FRCPC,** Associate Professor, Internal Medicine, Section of Physical Medicine and Rehabilitation, University of Manitoba, Manitoba, Canada; **Adrian J Farrell, BScHons, MBChB, MD, FRCP,** Consultant Rheumatology, WAHT (Worcestershire Acute Hospitals Trust), Worcestershire, United Kingdom; **Natasha Fernandes, MD, FRCPC** (Psychiatry), Ontario, Canada; **Nisha Fernandes, MD, FRCPC,** Ontario, Canada; **Catherine Ferrier, MD, CCFP (COE), FCFP,** Division of Geriatric Medicine, McGill University Health Centre, Quebec, Canada; **Baroness Finlay of Llandaff, FRCGP, FRCP, FMedSci, FHEA, FLSW,** London, United Kingdom; **Timothy Foggin, MD, BSc, MDCM, MPH, CCFP, FCFP, FRCPC, FACHE,** British Columbia, Canada; **Sarah Garside, MD, PhD, FRCPc,** Associate Professor, McMaster University, Ontario, Canada; **Sheenagh George, MD, FRCPC,** Nova Scotia, Canada; **Roger Ghoche, MDCM, CCFP-PC, MTS,** Rehabilitation and Palliative Care Medicine, Professeur Adjoint, Directeur de Cours de Méthode Clinique II, Université McGill, Assistant Professor, Course Director of Clinical Method II, McGill University, Mount Sinai Hospital, Montréal, Quebec, Canada; **Judy Glass, MD, FRCPC, DLFAPA,** Director, Emergency Psychiatry, Jewish General Hospital, Assistant Professor, McGill University, Department of Psychiatry, Québec, Canada; **John Good, MB, BCh, LM (Rotunda Hospital, Dublin), DRCOG (UK), MICGP (Ireland),** Past Medical Delegate for the International Committee of the Red Cross (Geneva, Switzerland), Ontario, Canada; **Randolph B Goossen, MD, CCFP, FCFP, FRCPC,** Psychiatry, Manitoba, Canada; **Michael Grasic, MD,** Family Medicine, Ontario, Canada; **Salvador M. Guinjoan, MD, PhD,** Oklahoma, USA; **Jack Haggarty, MD, FRCPC, CCPE,** Senior Medical Director, St. Joseph's Care Group, Professor and Chair, Section of Psychiatry, Northern Ont. School Medicine University, Ontario, Canada; **Lila Haj-Ahmad, MDCM,** Ontario, Canada; **Sheila Rutledge Harding, MD, MA, FRCPC,** Saskatchewan, Canada; **Margaret J. Hart, DO, FAAFP,** Family Medicine/Geriatric Medicine, Arizona, USA; **Philippa H. Hawley, FRCPC,** Clinical Professor Medical Director, Pain & Symptom Management/Palliative Care, BC, Cancer, British Columbia, Canada; **Kevin Hay, MRCPI, CCFP, FCFP,** Alberta, Canada; **J. David Henderson, MD, CCFP (PC),** Senior Medical Director Integrated Palliative Care Nova Scotia Health, Associate Professor Department of Family Medicine Dalhousie University, Nova Scotia, Canada; **Leonie Herx, MD, PhD, CCFP (PC), FCFP,** Palliative Medicine Consultant, Clinical Professor, Cumming School of Medicine, University of Calgary, Alberta, Canada; **Angela O Ho, HBSc, MD, FRCPC,** Assistant Professor, Dept of Psychiatry, Faculty of Medicine, University of Toronto, Ontario, Canada; **Sheila C Hollins, Professor the Baroness Hollins FRCPsych, FRCPCH, FRCP, FHEA,** Independent Life Peer, House of Lords, Westminster, UK, Emeritus Professor of Psychiatry, St George's, University of London, Past President, Royal College of Psychiatrists, Past President, British Medical Association, President, Royal College of Occupational Therapists, President, Royal Medical Benevolent Fund, London, United Kingdom; **Tae Young (Peter) Hong, MD, CFPC,** Ontario, Canada; **Philip Howard, MA, MD, GDL, LLM, FRCP,** Consultant Gastroenterologist, London, United Kingdom; **Trevor A. Hurwitz, MBChB, MRCP (UK), FRCP (C), Psychiatry FRCP (C)** Neurology, Diplomate in Psychiatry - American Board of Psychiatry and Neurology, Diplomate in Neurology - American Board of Psychiatry and Neurology, Diplomate Behavioral Neurology & Neuropsychiatry - United Council of Neurologic Subspecialties. Department of Psychiatry, University of British Columbia, British Columbia, Canada; **Lauren Jackson, BSc, MD, FCFP,** British Columbia, Canada; **Laila Jamal, MD, FRCPC,** Ontario, Canada; **Lawrence F. Jardine, BA, BMedSci, MD, FRCPC,** Honorary Consultant, Division of Pediatric Hematology Oncology, Children's Hospital (LHSC), Professor Emeritus (Pediatrics), Schulich School of Medicine and Dentistry, University of Western Ontario, Ontario, Canada; **Anna Jasinska, MD, FRCPC** (psychiatry), Pennsylvania, USA; **Will Johnston, MD,** Clinical assistant professor, Department of family practice, University of British Columbia, British Columbia, Canada; **Pamela Joseph, MD, FRCPC,** Ontario, Canada; **Stephanie Kafie, MD, CCFP (COE), BHsc,** Ontario, Canada; **Gurnaam Singh Kasbia, MD, BSc, FRCPC,** Psychiatrist, Ontario, Canada; **Ebru Kaya, MBBS, MRCP(UK),** Rose Family Chair in Palliative Medicine and Complex Care at University Health Network, Palliative Care Site Lead, Toronto General Hospital, Director, Division of Palliative Medicine, and Associate Professor, Department of Medicine, Temerty Faculty of Medicine, University of Toronto, President, Canadian Society of Palliative Care Physicians; **Eric Kelleher, MB, PhD, MRCP, MRCPsych,** Consultant Liaison Psychiatrist, Cork University Hospital & Mercy University Hospital, Honorary Clinical Senior Lecturer, University College Cork, Munster, Ireland; **Sister Nuala Kenny, OC, MD, FRCP(C),** Emeritus Professor Dalhousie University School of Medicine, Nova Scotia, Canada; **Laurence J. Kirmayer, MD, FRCPC, FCAHS, FRSC,** James McGill Professor & Director, Division of Social & Transcultural Psychiatry, McGill University, Quebec, Canada; **Leah Koetting, MD, CCFP,** Manitoba, Canada; **Mark S Komrad, MD, DFAPA,** Faculty of Psychiatry, Johns Hopkins, Tulane, and University of Maryland, Maryland, USA; **Jaro Kotalik, MD, FRCPC,** Ontario, Canada; **Jessica Kreviazuk, MD, FRCPC,** Assistant Professor University of Manitoba, Community Psychiatrist, Manitoba, Canada; Emeritus Professor **Linda Kristjanson, AO, PhD, FAICD, FTSE,** Victoria, Australia; **Mark Kristjanson, MD, CCFP,** Assistant Professor, Department of Family Medicine, Rady Faculty of Health Sciences, University of Manitoba, Manitoba, Canada; **Charles Lakusta, MD,** Alberta, Canada; **Karen Lam, MD, FRCPC,** Ontario, Canada; **Tim Lau, MD, FRCPC,** Associate Professor, University of Ottawa, Ontario, Canada; **Alejandro Lazo-Langner, MD, MSc, FRCPC,** Professor of Medicine, Oncology, and Epidemiology and Biostatistics, Chair/Chief, Division of Hematology, Department of Medicine, Western University, Hematology Division, London Health Sciences Centre, Ontario, Canada; **Mark Leakos, MD,** Saskatchewan, Canada; **Cassandra Lin-Pepe, MD, CCFP, Dip P Derm,** Ontario, Canada; **Jeffrey H Lipton, PhD, MD, FRCPC,** Clinician Investigator, Princess Margaret Cancer Centre, Cancer Clinical Research Unit (CCRU), Princess Margaret Cancer Centre, Professor of Medicine, University of Toronto, Ontario, Canada; **Iris Liu, MD,** British Columbia, Canada; **Matthew Loscalzo, LCSW, APOS Fellow,** Executive Director, People & Enterprise Transformation, Emeritus Professor Supportive Care Medicine, Professor Population Sciences, California, USA; **Joyce Lui, MD, CCFP,** Alberta, Canada; **Patrick MacGillivray, MD, CCFP,** Ontario, Canada; **James MacMillan, MD, CCFP,** Palliative Care, Saskatoon & Integrated North, Saskatchewan, Canada; **John Maher, MD, FRCPC,** President, Ontario Association for ACT & FACT, Editor-in-Chief, Journal of Ethics in Mental Health, Ontario, Canada; **Lauren Mai, MD, FRCPC,** Neurologist, Assistant Professor, Western University, Ontario, Canada; **Katalin Margittai, MD, FRCPC DABPN, DLFAPA,** Assistant Professor of Psychiatry, University of Toronto, Ontario, Canada; **Stephen Martin, MD, FCFP, FCBOM, ABPM(OM),** Quebec, Canada; **Karen Mason, MD,** Retired family physician, Ontario, Canada; **Paul McArthur, BSc, MD, CCFP,** Adjunct Professor of Family Medicine, University of Western Ontario, Ontario, Canada; **Annette McCarthy, MD, CCFP,** Family Physician, Newfoundland, Canada; **Gordon McDonald, MD, CCFP,** Department of Family Medicine, Dalhousie University, Fredericton, Canada; **C. Ann McKenzie, MD,** Manitoba, Canada; **Diane E. Meier, MD, FACP, FAAHPM,** Founder, Director Emerita and Strategic Medical Advisor, Center to Advance Palliative Care, Co-director, Patty and Jay Baker National Palliative Care Center, Professor, Department of Geriatrics and Palliative Medicine, Catherine Gaisman Professor of Medical Ethics, Icahn School of Medicine at Mount Sinai, New York, USA; **Tim Millea, MD,** Fellow, American Board of Orthopedic Surgeons, Iowa, USA; **Alisha Montes, MD, MPH, FRCP,** Pediatrician, Newfoundland, Canada; **José A. Morais, MD, FRCPC,** Director, Division of Geriatric Medicine, McGill University, Quebec, Canada; **Marie Angelle Moreau, MD,** General Internist, Alberta, Canada; **Louis Morissette, MD, FRCP,** Forensic Psychiatrist, UM, Institiut national de psychiatrie légale Philippe-Pinel, Quebec, Canada; **R. Sean Morrison, MD,** Ellen and Howard C. Katz Professor and Chair, Brookdale Department of Geriatrics and Palliative Medicine, Icahn School of Medicine at Mount Sinai, New York, USA; **Michael E. Mrozek, MD, FRCP(C),** Toronto, Ontario; **Christopher Newcombe, CCFP (EM),** British Columbia, Canada; **Nicholas Newman, MD, FRCSC,** Associate Professor, Department of Surgery, Université de Quebec, Quebec, Canada; **Laurence Normand-Rivest, MD,** Quebec, Canada; **Natalia Novosedlik, MD, CCFP(PC),** Ontario, Canada; **Kevin O'Kane, BSc(Hons), MB, ChB, LLM, FRCP, FRCPE,** Consultant in General & Acute Internal Medicine, London, United Kingdom; **Félix Pageau, MD, FRCPC, MA,** Philosophy, Geriatrician, Ethicist, Assistant Medicine Professor (Professeur adjoint médecin enseignant sous octoi), Department of Medicine, Faculty of Medicine, Laval University, Full researcher, VITAM - Research Center on Sustainable Health and the Centre d'excellence en vieillissement de Québec, Laval University and CIUSSS Capitale-National, Quebec, Canada; **Michael J. Passmore, MD, FRCPC,** British Columbia, Canada; **M. Jill Peacock, MD, FCFP** (retired), British Columbia, Canada; **José Pereira, MBChB, CCFP (PC), MSc, FCFP, PhD,** Professor, Division of Palliative Care, Department of Family Medicine, McMaster University, Ontario, Canada; **Donald J. Peters, MD, FRCPC** (retired), Manitoba, Canada; **Robin Pierucci, MD, MA,** Clinical Neonatologist, Michigan, USA; **Liette Pilon, MD,** Retired Family Practice, Quebec, Canada; **Ivan Poukhovski-Sheremetyev, MD, FRCPC,** Ontario, Canada; **François Primeau, MD, FRCPC,** Full Clinical Professor, Psychiatry and Neurosciences, Laval University, Québec, Canada; **Lukas Radbruch, MD, PHD,** Professor, Department of Palliative Medicine, University Hospital Bonn, Chair of the board of directors of IAHPC, North Rhine-Westphalia, Germany; **Larry Rados, MD, CCFP,** Manitoba, Canada; **Shirley Reddoch, MD, FAAP,** Pediatric Hematology/Oncology, Maryland, USA; **Claud Regnard, FRCP,** Honorary consultant in Palliative Care Medicine, St. Oswald's Hospice, Newcastle-upon-Tyne, United Kingdom; **Karen Reimers, MD, FRCPC,** Assistant Professor, University of Central Florida, Florida, USA; **Jean-Marie M. Ribeyre, MD, PhD, FRCPC,** Associate Professor/Professeur Agrege, St. Lawrence Valley Correctional and Treatment Centre - Secure Treatment Unit, Ontario, Canada; **Evan Ritchie, MD,** PGY-4, Anesthesiology & Pain Medicine, Alberta, Canada; **Roger Roberge, MD, PhD,** Quebec, Canada; **Anne Rose, MD,** Ontario, Canada; **Skye Rousseau, MD, FRCPC,** Assistant Professor, University of Manitoba, Dept. of Psychiatry, Manitoba, Canada; **Edward J Rzadki, BA, MD, FRCPC,** Assistant Professor Psychiatry U of T 1969 (retired), Past President, Ontario Psychiatric Association, Past Chief of Psychiatry, Etobicoke General Hospital, Past Chief of Staff, Etobicoke General Hospital, William Osler Health Centre, Past Corporate Chief of Psychiatry and Past Medical Director, Mental Health and Addictions, 1999-2007, Ontario, Canada; **Paul Saba, M.D. C.M. M.Sc.,** Family Physician & Author of Made to Live, Quebec, Canada; **Richard H. Sandler, MD,** Professor of Pediatrics, Professor of Mechanical and Aerospace Engineering, University of Central Florida, Florida, USA; **Jitender Sareen, MD,** Department Head, Psychiatry, University of Manitoba, Provincial Specialty Lead, Mental Health and Addictions, Shared Health, Manitoba, Canada; **Luke Savage, BKin(Hons), MD, CCFP, FCFP,** Alberta, Canada; **Anton Scamvougeras, MBChB, FRCPC,** Neuropsychiatrist, UBC Neuropsychiatry Program, Department of Psychiatry, University of British Columbia, British Columbia, Canada; **H. Schipper, BASc, MD, FRCP(C),** Professor of Medicine, Adjunct Professor of Law, University of Toronto, MindenSchipper & Associates Inc., Ontario, Canada; **Valerie Schulz, MD, FRCPC, MPH, CPC(HC),** Ontario, Canada; **John F Scott, MD,** Associate Professor, Palliative Medicine, University of Ottawa, Ontario, Canada; **Nathan Schaffer, MD, FRCPC,** Clinical Assistant Professor, Dept of Psychiatry, University of British Columbia; **Mary V. Seeman, OC, MDCM,** Department of Psychiatry, University of Toronto, Ontario, Canada; **Antonia Seli, BSc(Hons), MD, FRCPC,** Staff Psychiatrist, Adult Outpatient Services, Halton Healthcare - Oakville Trafalgar Memorial Hospital, Medical Lead - One Link, Assistant Clinical Professor - McMaster University, Ontario, Canada; **Keith Sequeira, MD, FRCPC,** Associate Professor, Schulich School of Medicine and Dentistry, Western University, Department of Physical Medicine and Rehabilitation, Ontario, Canada; **Amir Shamlou, MD, FRCPC,** Manitoba, Canada; **Alexander Simpson, MBChB, BMedSci, FRANZCP, FCPA,** Chair in Forensic Psychiatry, CAMH + University of Toronto, Senior Scientist, Research Program, CAMH, Professor, Department of Psychiatry, Temerty Faculty of Medicine, University of Toronto, Institute of Health Policy, Management and Evaluation, Dalla Lana School of Public Health - University of Toronto, Ontario, Canada; **Richard R. J. Smyth, MBBS, LRCP, FRCS, FRCSC** - retired, Adjunct Professor in Faculty of Science, Thompson Rivers University, BC, Clinical Instructor in Otolaryngology - Head and Neck Surgery, University of British Columbia, British Columbia, Canada; **Allan B. Steingart, MD, FRCPC,** Ontario, Canada; **Theresa Szezepaniak, MB ChB, DRCOG, Dip IMC(RCSEd),** LMCC, Hospitalist, Family Medicine, British Columbia, Canada; **Matthew David Swatski, MD,** University of Pittsburgh School of Medicine, SUNY at Buffalo Pediatrics Chief Resident, Pennsylvania, USA; **Sanasi Swatski, MD,** PGY2, Family Medicine, McMaster University, Ontario, Canada; **Sephora Tang, MD, FRCPC,** Assistant Professor, University of Ottawa, Ontario, Canada; **Agnes Tanguay, MD, CCFP,** Ontario, Canada; **Robert Twycross, DM, FRCP, FRCR,** Emeritus Clinical Reader in Palliative Medicine, Oxford University, Oxford, United Kingdom; **Stephen Vander Klippe,** Family Physician., Ontario, Canada; **Lilian Lee Yan Vivas, BSc, MD, FRCPC, CSCN, Dip. (EMG),** Physical Medicine and Rehabilitation, Ontario, Canada; **Lucas Vivas, CD, MD, MHSc (Bioethics), FRCPC,** Active Staff, General Internal Medicine, William Osler Health System, Assistant Clinical Professor (Adjunct), Department of Medicine, McMaster University, Ontario, Canada; **Declan Walsh, MD, MSc, FACP, FRCP(Edin),** Chair, Department of Supportive Oncology, Atrium Health Levine Cancer, Clinical Professor, Department of Internal Medicine, Wake Forest University School of Medicine, The Hemby Family Endowed Chair in Supportive Oncology, Editor-in-Chief, BMJ Supportive and Palliative Care, Fellow Emeritus, Trinity College, The University of Dublin, North Carolina, USA; **Ellen Warner, MD, MSc, FRCPC, FACP,** Division of Medical Oncology, Sunnybrook Odette Cancer Centre, Professor of Medicine, University of Toronto, Ontario, Canada; **Eric Wasylenko, MD, CCFP, (PC), MHSc (bioethics),** Adjunct, Division of Palliative Medicine, Department of Oncology, Cumming School of Medicine, University of Calgary; and Adjunct, John Dossetor Health Ethics Centre, University of Alberta, Alberta, Canada; **Shawn Whatley, MD, FCFP(EM),** Munk Senior Fellow in Health Policy, Macdonald-Laurier Institute, Past President, Ontario Medical Association, Ontario, Canada; **Frederick J. White, MD, FACC, FCCP, HEC-C,** Louisiana, USA; **DP Whitehouse, MB, ChB, MSc, PG Dip Pall Med,** Diploma Tropical Med and Hygiene, PG Cert International Development & Conflict, FRCP, AFOM., Consultant in Palliative, Respiratory and General Medicine., Bosham, United Kingdom; **James Wheeler, MD, MSC, CCFP, FCFP,** Ontario, Canada; **Kiely Williams, MD, CCFP, FCFP,** Alberta, Canada; **Maria Wolfs, MD, MHSc, FRCPC,** Ontario, Canada; **Jerome Yang, MD, MHSc, CCFP(AM),** British Columbia, Canada; **Jonathan Yang, MD, CCFP,** British Columbia, Canada; **Stanley Yaren, MD, FRCPC** (psychiatry), Associate Professor, University of Manitoba, Manitoba, Canada; **John W. S. Yun, MD, FRCPC,** Oncology, British Columbia, Canada; **Joel Zivot, MD, FRCPC,** Emory University, Georgia, USA; **Alexandra Zyla, MD,** PGY-2 Anatomical Pathology, University of Toronto, Ontario, Canada.

## Funding Information

No funding was received for this article.
